# Supplementary material for: Modeling glioblastoma heterogeneity as a dynamic network of cell states
Source: Mol Syst Biol. 2021 Sep 16;17(9):e10105. doi: 10.15252/msb.202010105 (PMC8444284; doi:10.15252/msb.202010105)
Supplement: Supplementary file 6 — Source Data for Figure 5 [file MSB-17-e10105-s004.zip › Figure5A_sourcedata/GSEA_3071/hallmarks_stateV.GseaPreranked.1623742546329/HALLMARK_APICAL_SURFACE.html]

Details for gene set HALLMARK\_APICAL\_SURFACE[GSEA]

|  || Dataset | state43071 |
| Phenotype | NoPhenotypeAvailable |
| Upregulated in class | na\_pos |
| GeneSet | HALLMARK\_APICAL\_SURFACE |
| Enrichment Score (ES) | 0.26644373 |
| Normalized Enrichment Score (NES) | 0.9122474 |
| Nominal p-value | 0.556962 |
| FDR q-value | 0.6041485 |
| FWER p-Value | 1.0 |
Table: GSEA Results Summary

  

Fig 1: Enrichment plot: HALLMARK\_APICAL\_SURFACE      
 Profile of the Running ES Score & Positions of GeneSet Members on the Rank Ordered List

  

| PROBE | GENE SYMBOL | GENE\_TITLE | RANK IN GENE LIST | RANK METRIC SCORE | RUNNING ES | CORE ENRICHMENT || 1 | BRCA1 |  |  | 46 | 1.592 | 0.1152 | Yes |
| 2 | GAS1 |  |  | 154 | 1.263 | 0.1932 | Yes |
| 3 | SULF2 |  |  | 236 | 1.131 | 0.2659 | Yes |
| 4 | APP |  |  | 570 | 0.818 | 0.2664 | Yes |
| 5 | FLOT2 |  |  | 879 | 0.668 | 0.2601 | No |
| 6 | SHROOM2 |  |  | 1431 | 0.497 | 0.1944 | No |
| 7 | ADIPOR2 |  |  | 2360 | 0.341 | 0.0451 | No |
| 8 | EPHB4 |  |  | 2559 | 0.315 | 0.0321 | No |
| 9 | SRPX |  |  | 2646 | 0.305 | 0.0395 | No |
| 10 | AKAP7 |  |  | 2858 | 0.284 | 0.0216 | No |
| 11 | NTNG1 |  |  | 3914 | -0.369 | -0.1495 | No |
| 12 | GSTM3 |  |  | 3966 | -0.381 | -0.1295 | No |
| 13 | GATA3 |  |  | 4015 | -0.392 | -0.1081 | No |
| 14 | ATP8B1 |  |  | 4161 | -0.427 | -0.1023 | No |
| 15 | THY1 |  |  | 4874 | -0.781 | -0.1764 | No |
| 16 | CRYBG1 |  |  | 4969 | -0.885 | -0.1253 | No |
| 17 | HSPB1 |  |  | 4992 | -0.920 | -0.0579 | No |
| 18 | PLAUR |  |  | 5185 | -1.479 | 0.0208 | No |
Table: GSEA details [plain text format]

  

Fig 2: HALLMARK\_APICAL\_SURFACE: Random ES distribution      
 Gene set null distribution of ES for **HALLMARK\_APICAL\_SURFACE**
